# Comparing virtual reality and simulation to teach the assessment and management of acute surgical scenarios: A pilot study

**DOI:** 10.1002/hsr2.2245

**Published:** 2024-07-08

**Authors:** Mi‐Tra Tran, Manal Ahmad, Kirtan Patel, Orestis Argyriou, Alun Davies, Joseph Shalhoub

**Affiliations:** ^1^ Faculty of Medicine Imperial College London London UK; ^2^ Department of Surgery and Cancer, Academic Section of Vascular Surgery Imperial College London London UK; ^3^ Imperial Vascular Unit Imperial College Healthcare NHS Trust London UK; ^4^ Department of General Surgery Imperial College Healthcare NHS Trust London UK

**Keywords:** education, mannequin, simulation, surgical, training, virtual reality

## Abstract

**Background and Aims:**

Traditional apprenticeship‐based surgical training presents with challenges, especially in acute scenarios. Simulation provides the current standard of facilitating surgical training in a low‐risk environment but is restricted by limited accessibility and high costs. Virtual reality (VR) offers immersive three‐dimensional computer‐generated training scenarios and can connect users from various locations. We aimed to compare the performance of junior doctors to manage an acute surgical scenario using VR and mannequin‐based simulation. We hypothesised that VR would be as effective as mannequin‐based simulation in performance outcomes.

**Methods:**

This multicentre, randomised controlled pilot study was conducted with eighteen junior doctor volunteers (Foundation and Core Trainee Year 1). Ten were randomly allocated to VR and eight to mannequin‐based simulation. Participants completed questionnaires and a 15‐min pneumothorax scenario. Quantitative metrics included overall score, time‐to‐critical decisions, and academic buoyancy scores (ABS). Qualitative metrics included participants' likes and dislikes of their allocated simulation modality.

**Results:**

VR participants scored significantly higher than mannequin‐based simulation participants in overall scores (74.30% (SD ± 5.08%) *vs.* 59.75% (SD ± 10.14) (*p* = 0.04)), and technical skills aspects (77.20% (SD ± 8.01%) *vs.* 65.00% (SD ± 8.21%) (*p* = 0.01)). Mannequin‐based simulation participants initiated critical decisions faster and demonstrated a trend towards a faster mean time‐to‐completion (*p* = 0.06). ABS scores increased for both study groups, though was only significant for VR participants (*p* ≤ 0.01). VR participants liked how VR fostered independent learning but disliked the formulaic content and impaired communication‐learning compared to mannequin‐based simulation.

**Conclusion:**

Both VR and mannequin‐based simulation training are effective in training junior doctors in acute surgical scenarios but present different educational benefits. Future research should recruit a larger sample size for a full comparative randomised controlled trial.

## 1 BACKGROUND

Surgical training is historically an arduous journey. Traditionally, surgical procedures were taught using the apprenticeship‐based Halstedian model of “see one, do one, teach one".^1^, ^2^ Whilst this fosters a peer‐assisted learning culture, it is criticized by concerns to patient safety as trainees, who may lack the skills, practice procedures on real patients. Further factors such as time constraints and high‐pressure environments make the operating theatre unsuitable as a classroom for high‐risk, acute surgeries.

Up to 42% of residents feel inadequately trained to safely perform various procedures unassisted for the first time, although the true prevalence may be higher.^3^, ^4^ Halstead's model is also restricted by the 48‐h work week,^5^ which whilst implemented for health and safety, these shorter hours make sourcing additional time for surgical exposure alongside numerous clinical demands difficult.^6^ Furthermore, the COVID‐19 pandemic negatively impacted surgical training opportunities, with progression within training affected by cancellations of elective procedures and redeployment of surgical trainees.^7^ These negative effects were felt globally, with burnout rates amongst surgical trainees peaking as high as 95.2%.^8^

In recent years, the surgical curriculum has evolved from a numbers‐based mindset which focuses on counting how many procedures a trainee has performed, to a competency‐based mindset where trainees are evaluated on what procedures they can perform independently and how well they can do so. This shifts the training approach from assuming that exposure to procedures over a fixed timeframe will be sufficient to achieve competency, to a more encompassing approach which ensures that trainees are obtaining the knowledge and skills needed to become competent.^9^ Considering different ways trainees learn helps to effectively inform teaching strategies, especially as new surgical techniques and learning modalities are continually being implemented in the surgical curriculum.

Simulation is a widely used and validated form of surgical training which provides safe and structured opportunities to practice skills outside the operating theatre. However, hiring equipment, space and personnel needed to run simulations can be expensive. Medical school simulation examinations can cost greater than £355 per student.^10^ Cadaver‐based simulation is especially costly, considering the limited supply and storage.^11^ Furthermore, simulation is usually group‐based, requiring trainees to be physically present at the same time. The time‐consuming efforts required to travel to different training centres makes in‐person simulation less accessible to those located in rural areas.

Virtual reality (VR) offers a significant advance for surgical simulation. VR is a computer‐generated environment, where individuals can interact with a fully immersed three dimensional virtual world through a head‐mounted display.^12^ Having been popularised by the gaming industry, the cost of consumer‐grade headsets has become more affordable.^13^ VR has been used previously by the aviation sector for flight simulation,^14^ and military sectors for replicating battlefield scenarios.^15^ Its portability allows teaching to be conducted in convenient spaces, whilst also connecting multiple users across different locations, saving on time and travel.^16^, ^17^ High‐fidelity VR simulators can be very realistic, and developers can tailor experiences with various complexity levels. Furthermore, VR is more cost‐efficient than other simulation modalities requiring fewer resources to operate, and is easier to scale and distribute.^18^, ^19^, ^20^

Favourable outcomes from VR‐based training have been demonstrated. Harrington et al. and Colonna et al. both demonstrated that their trauma simulators were able to distinguish decision‐making abilities between trainees of different levels, with higher scores achieved by those with more training experience.^21^, ^22^ Kiyozumi et al. showed that their VR pre‐hospital trauma evaluation exercises allowed completion of more training scenarios in a shorter amount of time, compared to standard face‐to‐face training, whilst being able to facilitate competency in initial assessment procedures.^23^

Outcomes of simulation can be broadly divided into technical or nontechnical skills acquisition. Technical skills in trauma involves interventions such as chest drain insertions and pericardiocentesis, whilst nontechnical skills involve leadership and management.^24^ Such skills can be examined in standardised training courses, like the ubiquitous Advanced Trauma Life Support (ATLS) course, which has reformed trauma management in more than 60 countries.^25^ Examinations are commonly assessed using Objective Structured Clinical Examination (OSCE) frameworks, whereby candidates are objectively marked against standardised scoring sheets whilst completing timed activities in simulated stations.^26^

During simulation, stress and self‐perceived poor task execution may hinder trainee performance. Academic buoyancy may protect from the effects of psychological distress.^27^, ^28^ The academic buoyancy scale (ABS) is a validated scoring system created by Marsh et al. which reflects a learner's ability to successfully deal with short‐term, minor academic setbacks, such as poor grades and exam pressures.^27^ It consists of four questions on a 7‐point Likert scale; learners who rate themselves with higher scores reflect higher academic buoyancy, which can translate into increased long‐term resilience (Supporting Information S1: Appendix S1). Analysing simulation results in the context of ABS scores may help to better understand overall performance.

### 1.1 Aim

VR is not yet a validated training modality compared to simulation, being a more recent development. This pilot study aimed to compare the performance of junior doctors to assess and manage an acute surgical scenario using VR and mannequin‐based OSCE simulation. We analysed participants' objective performance metrics, subjective perceptions on their approach to the scenario, and any correlations between ABS and OSCE scores. Furthermore, we evaluated feasibility for a subsequent full comparative randomised controlled trial.

We hypothesised VR would be as effective as mannequin‐based simulators in the assessment and management of acute surgical scenarios.

## 2 METHODS

This was a multicentre, randomised controlled pilot study conducted across two London hospital sites between March 2023 and May 2023. Recruitment emails and posters were distributed amongst Foundation Year 1 and 2 (FY1/FY2) and Year 1 Core Surgical Trainees (CST). Exclusion criteria involved previous ATLS course attendance to ensure similar educational maturity of participants.

**Figure 1:**
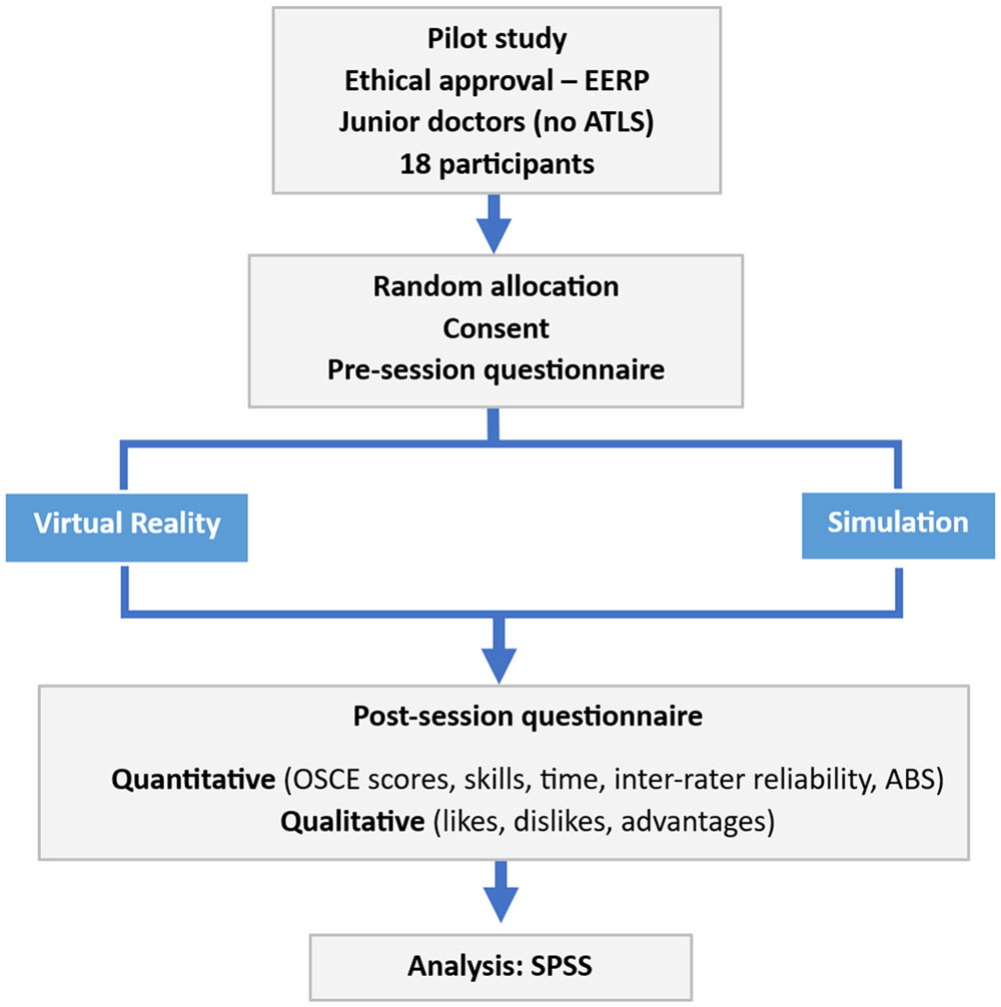
Consort diagram.

Eighteen volunteer participants were recruited and randomised to either Simulation or VR (Figure 1), where the first 10 participants to volunteer were allocated to VR and the remainder to Simulation. Participants were allocated a study number to de‐identify their data. All participants provided written informed consent and could withdraw their data anytime until the point of statistical analysis, after which it would be difficult to separate the deidentified data. Basic demographics including age, grade and previous exposure to surgical specialities were recorded. Ethical approval was granted by the Education Ethics Review Process board at Imperial College London.

The Oculus Meta Quest 2 (Facebook, Meta Platforms, Inc.)^29^ was used for VR simulation, featuring an immersive 360° view*.* The display was casted onto a secondary monitor to simultaneously observe the participants' viewpoints. The Oxford Medical Simulation (OMS) software application was used with licence agreement.^30^ VR participants undertook a 30‐min VR tutorial to familiarise themselves with the equipment and navigation.

The time‐limited 15‐min VR acute surgical scenario featured a pneumothorax case, where an acutely tachypnoeic patient presented on a background of chronic obstructive pulmonary disease. Management of pneumothorax is part of the ATLS curriculum and Intercollegiate Surgical Curriculum Programme for core surgical training. A virtual nurse was present as part of the scenario to assist the participant. The OMS case profile and learning objectives are outlined in Supporting Information S1: Appendix S2 *.* Participants were tasked to assess and manage the patient, and possible actions were presented in a drop‐down list (Supporting Information S1: Appendix S3)*.* Following the scenario, OMS provided feedback on areas of good performance, improvement, and an overall score.

An in‐person 15‐min mannequin‐based simulation (‘Simulation’) OSCE was adapted using the same pneumothorax scenario, where participants were tasked to independently assess and manage the patient. An in‐depth Simulation tutorial was not necessary as participants already had simulation exposure from their undergraduate and Foundation Year training. Participants were instead introduced to the simulation room and shown where relevant instruments were located, like in the VR tutorial. A Simulation marking scheme was created following OMS and OSCE marking frameworks (Supporting Information S1: Appendix S4). Two human assessors, who hold ATLS qualifications and interests in medical education, were used for Simulation to reduce grading bias and assessed the participants independently from each other.

All participants completed a pre‐teaching questionnaire to assess their confidence in managing acute surgical scenarios, and any previous simulation or VR exposure. Post‐teaching questionnaires were also completed. Performance metrics were categorised into quantitative and qualitative measures*.*

Quantitative measures:

1. Overall OSCE simulation scores
2. Skills domains scores: Communication, Nontechnical, Teamwork and Technical aspects, as pre‐determined by OMS (Supporting Information S1: Appendix S5)
3. Scenario completion time and time‐to‐critical decisions, as pre‐determined by OMS
4. Likert scale ratings of participants' preparedness and confidence of the scenario
5. Pre‐scenario and post‐scenario ABS scores

Qualitative measures:

1. Whether participants would like to be taught using their allocated modality again
2. Likes and dislikes about their allocated teaching modality
3. Advantages or disadvantages of their allocated modality

Statistical analysis was performed using Statistical Product and Service Solutions (IBM Corp. 2020. IBM SPSS Statistics for Windows, Version 27.0). The Shapiro‐Wilk test was used to assess normality. The Independent T‐Test and Mann–Whitney U Test compared overall OSCE and skills domains scores, and time‐to‐critical decisions. The paired T‐test and Wilcoxin signed rank test compared ABS scores. Pearson Correlation Coefficient evaluated any correlation between overall OSCE and ABS scores. Intraclass Correlation Coefficient calculated inter‐rater reliability between the human Simulation assessors. A *p*‐value < 0.05 was considered statistically significant. A power calculation was not considered necessary for this pilot study.

## 3 RESULTS

Eighteen junior doctors were recruited with nine FY1, five FY2, and four CST (Table 1). Ten participants were allocated to VR and eight to Simulation. The mean age was 26.0 years (SD ± 2.2 years), with eleven males and seven females. Most participants had a general or vascular surgery background, were interested in acute surgical management, had undertaken previous simulation‐based courses and had not been part of a trauma assessment team. Most had not used the Oculus headset and, of those who had, was primarily for gaming purposes.

**Table 1:** Demographic data of participants.

Demographic data			
	Overall (*n* = 18)	VR (*n* = 10)	Simulation (*n* = 8)
**Gender**			
Male	11	5	6
Female	7	5	2
**Age**			
	26.0 ± 2.2	25.9 ± 2.1	27.0 ± 2.5
**Handedness**			
Right	15	8	7
Left	3	2	1
**Grade**			
Foundation year 1	9	5	4
Foundation year 2	5	4	1
Core trainee	4	1	3
**Interested in acute surgical management scenarios?**			
Yes	17	10	7
No	1	**‐**	1
a **Undertaken previous BLS/ALS/ALERT/ATLS courses?**			
ALS	10	7	3
BLS	7	5	2
ILS	3	1	2
Unspecified	3	**‐**	3
**Undertaken previous simulation‐based courses?**			
Yes	15	9	6
No	3	1	2
**Previously been part of a trauma assessment team?**			
Yes	6	3	3
No	12	7	5
**Previously worked in any specialities?**			
Accident & emergency	5	3	2
Anaesthetics/Intensive therapy	2	1	1
General surgery	7	3	4
Major trauma	1	**‐**	1
Trauma & orthopaedics	7	2	5
Vascular surgery	4	3	1
**Currently working in any specialities?**			
Accident & emergency	1	1	**‐**
Anaesthetics/Intensive therapy	1	1	**‐**
General surgery	3	1	2
Major trauma	2	1	1
Trauma & orthopaedics	2	**‐**	2
Vascular surgery	5	4	1
**Previously used Oculus headset?**			
Yes	5	4	1
No	13	6	7
**Used headset for what reason?**			
Gaming	4	3	1
Medical education	2	2	‐
**Any reason why you cannot use Oculus headset?**			
Yes	‐	‐	‐
No	18	10	8

*Note*: Age is presented as (Mean ± Standard Deviation).

a Abbreviations: ALERT, Acute Life Threatening Events Recognition and Treatment; ALS, Adult Life Support; ATLS, Advanced Trauma and Life Support; BLS, Basic Life Support.

### 3.1 Quantitative analysis

Participants' self‐rated confidence scores in the assessment and management of acute surgical scenarios improved from 4.56 out of 10.00 (SD ± 1.69) pre‐session to 7.56 (SD ± 0.92) post‐session (Tables 2a and 2b).

**Table 2a:** Preparedness and confidence of the early assessment and management of acute surgical scenarios before the session.

Pre‐teaching data						
	Overall		VR		Simulation	
	Mean	SD	Mean	SD	Mean	SD
How prepared do you feel in the early assessment and management of acute surgical scenarios?	4.61	1.75	5.00	1.89	4.13	1.55
How confident do you feel in the early assessment and management of acute surgical scenarios?	4.56	1.69	4.90	1.79	4.13	1.55

*Note*: Rating on a scale of 1–10, with 1 being least and 10 being most. Reported as mean and standard deviation (SD).

**Table 2b:** Improvement and confidence of the early assessment and management of acute surgical scenarios following the session.

Post‐teaching data						
	Overall		VR		Simulation	
	Mean	SD	Mean	SD	Mean	SD
How much do you think you have improved?	6.83	1.29	6.60	1.43	7.13	1.13
How confident do you feel in the early assessment and management of acute surgical scenarios?	7.56	0.92	7.40	0.70	7.75	1.16

*Note*: Rating on a scale of 1–10, with 1 being least and 10 being most. Reported as mean and standard deviation (SD).

VR participants had a higher mean post‐session score compared to Simulation participants (74.30% (SD ± 5.08%) vs. 59.75% (SD ± 10.14%) (*p* = 0.04). Of the skills domains, VR participants scored higher in all four domains except nontechnical aspects. Significance was only reached for technical aspects (77.20% (SD ± 8.01%) *vs.* 65.00% (SD ± 8.21%), *p* = 0.01) (Table 3). These results are graphically represented in Supporting Information S1: Appendix S6.

**Table 3:** Comparison of Objective Structured Clinical Examination scores and skills domains.

Post‐session results							
	Overall		VR		Simulation		Significance
	Mean	SD	Mean	SD	Mean	SD	
Post‐session score	67.83%	10.56	74.30%	5.08	59.75%	10.14	*p* = 0.04*
Communication	64.33%	24.67	74.70%	11.65	51.38%	30.96	*p* = 0.07
Nontechnical	50.00%	25.72	45.00%	28.38	56.25%	22.16	*p* = 0.46
Teamwork	81.11%	12.78	84.00%	12.65	77.50%	12.82	*p* = 0.36
Technical	71.78%	10.03	77.20%	8.01	65.00%	8.21	*p* = 0.01**

*Note*: Significance is calculated between VR and simulation scores. Reported as mean and standard deviation (SD). An asterisk denotes p‐value is significant, whereby * means *p* ≤ 0.05, and ** means *p* ≤ 0.01.

Simulation participants had a trend towards a shorter mean scenario completion time of 11:51 min (SD ± 03:04 min) compared to 13:46 min (SD ± 02:14 min) (*p* = 0.06). They were also quicker to initiate all critical decisions, which were statistically significant for the actions: “assess airway” (*p* = 0.01), “auscultate chest” (*p* < 0.001), “request chest x‐ray” (*p* < 0.001), and “insert chest drain” (*p* = 0.01) (Table 4). These results are graphically represented in Supporting Information S1: Appendix S7.

**Table 4:** Comparison of scenario completion time and time‐to‐critical decision.

	Time‐to‐critical decision (min)				
	VR		Simulation		Significance
Critical decision	Mean	SD	Mean	SD	
Continuous monitoring	01:28	01:32	01:05	00:45	*p* = 1.00
Oxygen delivery	01:55	01:29	01:49	01:23	*p* = 0.63
Verify name and date of birth	‐	‐	‐	‐	‐
Assess airway	04:08	01:05	02:01	01:37	*p* = 0.01**
Auscultate chest	05:58	01:23	02:10	01:28	*p* ≤ 0.001***
Request chest X‐ray	07:36	01:48	02:56	01:06	*p* ≤ 0.001***
Insert chest drain	10:02	02:47	06:13	02:26	*p* = 0.01**
Time efficiency	13:46	02:14	11:51	03:04	*p* = 0.06

*Note*: Reported as mean and standard deviation (SD). An asterisk denotes p‐value is significant, whereby ** means *p* ≤ 0.01 and *** means *p* ≤ 0.001. No mean or standard deviation (SD) available for “verify name and date of birth” as too few participants performed the action. Critical decisions not performed by some participants were classed as missing data. One virtual reality (VR) participant did not perform a chest drain, three VR participants did not assess the airway. Seven VR and three Simulation participants did not verify the patient's name and date of birth.

Both VR and Simulation groups saw an overall increase in post‐scenario ABS scores, with the mean VR ABS score increasing from 17.50 (SD ± 2.88) to 19.10 (SD ± 3.18), and from 17.25 (SD ± 5.60) to 17.75 (SD ± 4.98) for Simulation, though this increase was only significant for VR (*p* = 0.01) (Table 5). When ABS scores were compared with OSCE scores, no significant correlation was found, with an r‐value of 0.02 (*p* = 0.94). These results are graphically represented in Supporting Information S1: Appendix S8.

**Table 5:** Comparison of pre‐ABS and post‐ABS scores.

ABS scores										
	VR					Simulation				
	Pre		Post		Significance	Pre		Post		Significance
	Mean	SD	Mean	SD		Mean	SD	Mean	SD	
Overall ABS score	17.50	2.88	19.10	3.18	*p* = 0.01**	17.25	5.60	17.75	4.98	*p* = 0.28
I don't let study stress get on top of me	4.30	1.06	4.50	1.27	*p* = 0.51	3.88	1.46	4.00	1.51	*p* = 0.35
I think I'm good at dealing with schoolwork pressures	5.10	0.99	5.30	0.95	*p* = 0.16	4.75	0.89	4.75	1.16	p = 1.00
I don't let a bad mark affect my confidence	3.40	1.17	4.30	0.95	*p* = 0.07	3.88	1.55	4.13	1.55	*p* = 0.32
I'm good at dealing with setbacks	4.70	1.06	5.00	1.15	*p* = 0.41	4.75	1.28	4.88	1.25	*p* = 0.32

*Note*: Reported as mean and standard deviation (SD). An asterisk denotes p‐value is significant, whereby ** means *p* ≤ 0.01.

The two assessors' scores for Simulation marking were very similar with mean scores of 60.00% (SD ± 0.10%) and 59.00% (SD ± 0.10%). The Intraclass Correlation Coefficient was 0.96 (95% CI [0.83, 0.99]), whereby a coefficient greater than 0.90 indicates excellent inter‐rater reliability.^31^

### 3.2 Qualitative analysis

Nine of 10 VR participants would like to have the option of being taught using VR in the future, with the remaining participant stating “in conjunction with simulation.” All Simulation participants would like to have the option of being taught using simulation in future.

What participants liked and disliked about their simulation modality are presented in Table 6a, categorised by common themes.

**Table 6a:** Likes and dislikes about virtual reality and Simulation.

Theme	Likes about VR
Safe setting	“Felt like a safe and supported setting, not stressed about making mistakes.” “Did not worry about making mistakes”
Realistic	“Felt very realistic in terms of ordering and viewing investigations” “Very realistic. In some elements, more realistic than sim[ulation]” “Felt quite real” “Very close to real life” “Very immersive”
Interactive	“It was very interactive and the scenarios were engaging” “Remembering all the aspects/information available to you and allocation of duties when the options are available in the drop‐down menu”
Independent learning	“Can continue practising scenarios in which participants individually lack confidence” “Can repeat sessions in own time if desired” “Can go through scenarios in own time” “Once you practice, you can really get comfortable with a range of scenarios before seeing them on the ward. I found it really beneficial to myself as a visual learner and wish there were more opportunities to learn via this method”
Resource‐efficient	“Very effective form of self‐directed learning, especially if other people are not available to run sim[ulation] sessions ‐ which can be quite resource‐intensive” “Efficient teaching method requiring less manpower”
Feedback	“Great to get feedback straight after” “Allows students to get immediate feedback” “Methodical mark system at end”

Theme	Dislikes about VR
Adjustment	“Takes some time to get used to the headset and the programme. However, the gain to knowledge over time if you are given lots of exposure to this form of learning” “A little disorientating at first” “VR did not let me do what I wanted and I couldn't talk to the patient or do activities simultaneously” “I also felt that some of the options were slightly unrealistic”
Technical	“1 glitch on the programme but minimal impact to learning” “Lack of peripheral vision” “More difficult to locate visual prompts” “Headset is slightly bulky and can be uncomfortable if wearing spectacles.”
Communication	“In real simulation, it is helpful having a team member in person that you can verbally communicate to and think aloud” “Lack of communication” “Not able to speak to the patient” “Communication learning may be slightly impaired as the scenario phrases questions for you”
Content structure	“Tick box” “More formulaic, with specific options of management already laid out in front of you” “In real life and in previous simulation training I've done, it's been less prescriptive and directed” “It also gave me options rather than let me decide what I wanted to do spontaneously”
Practical skills	“Does not help assess the practical skills e.g. putting in a chest drain, doing an ABG [arterial blood gas] etc.”

Theme	Likes about mannequin‐based simulation
Reflects real‐life	“Practicing acute scenarios is essential for real‐life situations” “Allows you to feel the pressure of real‐life scenarios”
Natural	“More natural, easier to make decisions and do things following the natural flow” “More relaxed” “More freedom to go off‐piste and still get to the answer”
Interactive	“Hands on, requires communication and practicing those skills” “Sim[ulations] are good to get confident in the practice of communication and managing under pressure” “Interactive learning” “Engaging” “It is very interactive”

Theme	Dislikes about mannequin‐based simulation
Unrealistic	“Sim[ulation] does not reflect a real patient, therefore harder.” “Sometimes not realistic/low fidelity because you don't actually take the bloods etc” “Unable to physically examine a patient”

Similarly, common perceived advantages of participants' simulation modality are presented in Table 6b, categorised by common themes.

**Table 6b:** Perceived advantages of virtual reality and simulation.

Theme	Advantages of VR over other teaching methods
Safe setting	“Can be more self‐directed, can be better for students/participants who are not confident/loud in group sim[ulation] settings” “Feels like a safe space to make mistakes and learn” “More comfortable with less of an audience.” “Less stress than SIM session as a group.” “Gives hospital exposure in a safe environment.” “Feels safe and supported, less worried about making mistakes as real simulation can sometimes feel intimidating”
Efficient	“Efficient teaching method requiring less manpower” “More efficient”. “Immediate response and no issues or delays with mannequins, telephone calls or imaging requests” “Potentially cheaper and less resource heavy than traditional in‐person simulation” “Relatively resource cheap compared to SIM rooms and staffing required to run sessions.”
Feedback	“Allows students to get immediate feedback” “Useful to see clear feedback points regarding what I did well and what I missed, and the timing of each step.”
Independent learning	“Can repeat sessions in own time if desired”. “Can implement a huge number of scenarios and promote independent learning.” “Great for visual learners. Great for repetition of acute scenarios and building confidence. Able to practice more in own time.”

Theme	Advantages of mannequin‐based simulation over other teaching methods
More skills targeted	“enforces more reflective practice” “Useful to feel like you are in the space and communicating, and also able to practice with a mannikin”
Unsupervised practice	“allows me to practice sims outside a supervised environment, sims are really rare in my med[ical] school teaching”
Memorable	“Hands on teaching is more memorable and life‐like than textbook learning.”
Familiar modality	“used it previously”

## 4 DISCUSSION

In summary, VR participants had a significantly higher overall OSCE score compared to Simulation participants and scored significantly higher in technical aspects. Simulation participants initiated critical decisions and completed the scenario faster, although faster completion times do not necessarily equate to having achieved competence. VR participants also had significantly improved ABS scores, suggesting that having access to the VR teaching reinforced their academic buoyancy which, over a period of time, can translate to resilience.

Whilst ABS scores improved in the VR group more than in Simulation, though they did not correlate with the overall marks, it supports the proposition that VR may provide a more comfortable and self‐directed environment for participants to hone their clinical skills. Meanwhile, Simulation could be perceived as more stressful due to the in‐person presence and direct observers, which could have contributed to their lower ABS scores. In clinical practice, trainees are not being observed to the same extent as in a simulated examination, allowing VR training to more reflective of the working environment. Nonetheless, stress is inevitable in acute surgical training so it would benefit trainees to learn ways to manage stress in practice scenarios. From qualitative feedback, it is clear that VR is favoured in terms of its setting, efficiency and accessibility, and offers a well‐received adjunct to learning to complement existing methods or for sole use where time, financial and geographical factors can otherwise hinder accessibility.

The options available in the drop‐down menu provide prompts for VR participants to choose actions from which could make the scenario easier and too formulaic. It also hinders the ability to multitask, whereby VR participants could only execute one action at a time, whilst Simulation participants could, for example, take a history and examine the patient simultaneously. This fosters their communication skills unlike in VR whereby the scenario phrases questions for participants. Multitasking in Simulation could explain why participants had faster critical action and scenario completion times. Notably, Simulation participants requested a chest drain nearly 4 min faster on average than VR participants. This is clinically important where a chest drain should be promptly inserted in a tension pneumothorax compared to a simple spontaneous pneumothorax. Bias could exist within the Simulation group which consisted of more experienced CST, thus also resulting in faster action times.

Prompting from the VR drop‐down menu options could have also contributed to VR participants' higher OSCE scores and technical aspects. For example, “asking for allergies” was performed in 90% of VR participants, where it was presented as an option to choose from, compared to 38% in Simulation where no overt prompt was provided. Furthermore, prompting was seen when VR participants “phoned the senior.” In one case, a participant had missed the pneumothorax signs on the chest radiograph, but the senior on‐call explained the next steps of management assuming the participant had correctly diagnosed the patient.

Despite an excellent inter‐rater reliability between the two human Simulation assessors, they seemed to be more critical in marking compared to the VR marking framework. For example, the VR software awarded participants a mark for choosing the action of “continuous monitoring,” whereas Simulation assessors were hesitant to award marks if participants did not indicate monitoring needed to be “continuous.” This may be attributed to insight into the clinical aspects which are not necessarily translated to virtual reality.

Performing “Hand hygiene” was a poorly scored action, with only two VR participants doing so and none in Simulation. In VR, this could be due to the scenario starting with the participant facing the patient with the sink for hand hygiene directly behind them, removing the visual prompt. However, this visual prompt was present in Simulation as a box of clinical gloves by the bedside. Participants could have viewed the mannequin as an unrealistic, low‐fidelity model and therefore did not immediately register to wear gloves. Similarly, verifying the patient's details was performed by only one VR participant, possibly because the nurse introduces the patient's name already, whereas five Simulation participants verified the patient perhaps because this interaction presents more naturally in real‐life.

A study by Proctor et al. which compared the effectiveness between VR and mannequin‐based simulation for a surgical cricothyroidotomy scenario supports this study.^32^ Both modalities met training task performance evaluation requirements by participants, and met 95% user technology acceptance and 85% user recommendation levels. It was suggested that either modality was able to complement, reduce or replace other training modalities, such as cadaver models.

In contrast, a systematic review by Mackenzie et al.^33^ found there was inadequate evidence to suggest that VR can facilitate training for open trauma surgery or replace cadavers. This was mainly attributed to poor study designs and low sample sizes, which most studies were subject to, the latter of which applies to this present study. However, it is worth noting that training to manage an ATLS scenario is markedly different from training for open trauma surgery.

This study's strengths lie in contributing to a small pool of literature, as there is a paucity of research directly comparing the use of VR and Simulation to train doctors in acute surgical scenarios. It is also the first to look at ABS changes in VR for surgical teaching. Bias was reduced where possible. The study has demonstrated excellent inter‐rater reliability in marking Simulation scenarios. Grading bias was eliminated in VR which automatically provides a score based on the actions performed. Furthermore, the questionnaires contained sections for free‐text feedback to reduce acquiescence bias, and feedback was anonymized to reduce courtesy bias.

The limitations of this study include a small and slightly unequal sample size, collected using convenience sampling, meaning the results are not necessarily generalisable. The method of randomly allocating participants on a *first‐come first‐served* basis was used to overcome the logistical difficulties encountered during recruitment. There were few convenient times where doctors were available to convene on a specific date outside scheduled working hours which also coincided with availability of the Simulation suite, hence the lack of Simulation participants. Therefore, this method of “randomization” allowed data collection for the VR cohort to begin before volunteers became unavailable, to prevent delays in data collection, whilst recruitment for the Simulation cohort was ongoing. Inadvertently, this highlights the convenience and portability of VR as a teaching modality. There is also inherit self‐selection bias within this volunteer sample towards learners and acute surgical speciality trainees. Furthermore, the more subjective critical nature of the Simulation marking, as previously explained, could have contributed to the Simulation participants' lower scores overall, making it an inaccurate reflection of the VR group's relative performance.

Future studies could recruit a larger sample size, with scope for a priori sub‐group analyses between novices and advanced clinicians to examine the construct validity of the VR software. Investigating the degree to which VR‐based training stimulates meaningful learning which translates into real‐world performance benefits is important. Transfer quality, which reflects effective learning, would ideally involve evaluation of skills and competences in the actual clinical setting after VR training. Indirect predictors of skills transfer include measurement of simulation validity, that is whether accurate and immersive conditions of real‐world scenarios are replicated, engagement of learning strategies and retention of learning outcomes. Research should also establish whether spaced VR teaching sessions translate to improved long‐term markers of resilience. A cost‐effectiveness analysis between VR and Simulation would be useful for educational providers and could provide insight into use in low‐resource settings. Areas to investigate include initial starting costs, scenario development, and maintaining internet access.

## 5 CONCLUSION

In this randomised controlled pilot trial of 18 participants, VR participants scored significantly higher than Simulation participants during an acute surgical scenario, whilst Simulation participants initiated critical decisions and completed the scenario faster. VR benefits by allowing off‐site training and improves short‐term markers of confidence. Where VR prevails in aspects such as fostering independent learning and allowing immediate feedback, it lacks elements of what Simulation provides participants with, including the opportunity to practice communication skills and make clinical decisions following a more natural flow. Overall, both VR and mannequin‐based simulation training methods are effective educational modalities which can be used to train junior doctors in acute surgical scenarios but present different educational benefits. Future research should conduct a full comparative randomised control trial with a larger sample size and perform a cost‐effective analysis.

## AUTHOR CONTRIBUTIONS

**Mi‐Tra Tran**: Data curation; formal analysis; investigation; methodology; project administration; writing—original draft; writing—review and editing. **Manal Ahmad**: Conceptualization; data curation; formal analysis; funding acquisition; investigation; methodology; project administration; resources; supervision; writing—review and editing. **Kirtan Patel**: Data curation; investigation. **Orestis Argyriou**: Data curation; investigation. **Alun Davies**: Conceptualization; supervision; writing—review and editing. **Joseph Shalhoub**: Conceptualization; supervision; writing—review and editing.

## CONFLICT OF INTEREST STATEMENT

The authors declare no conflict of interest.

## ETHICS APPROVAL AND CONSENT TO PARTICIPATE

Ethical approval was granted by the Education Ethics Review Process board at Imperial College London.

## TRANSPARENCY STATEMENT

The lead author Mi‐Tra Tran affirms that this manuscript is an honest, accurate, and transparent account of the study being reported; that no important aspects of the study have been omitted; and that any discrepancies from the study as planned (and, if relevant, registered) have been explained.

## Supporting information

Supporting information.

## Data Availability

The authors confirm that the data supporting the findings of this study are available within the article and supplementary materials.
