# Supplementary material for: Comparing virtual reality and simulation to teach the assessment and management of acute surgical scenarios: A pilot study
Source: Health Sci Rep. 2024 Jul 8;7(7):e2245. doi: 10.1002/hsr2.2245 (PMC11231037; doi:10.1002/hsr2.2245)

**Appendix:**

Appendix 1 – Academic Buoyancy Scale

Appendix 2 – Case profile and learning objectives

Appendix 3 – VR scenario

Appendix 4 – Marking scheme

Appendix 5 – Actions categorised by skills domains

Appendix 6 – Graphical representations of OSCE scores and Skills domains

Appendix 7 – Graphical representation of time-to-critical decision and completion

Appendix 8 – Graphical representations of ABS scores

**Appendix 1 – Academic Buoyancy Scale**

The Academic Buoyancy Scale is shown with 4 questions, rated using a 7-point Likert scale, with 1 being strongly disagree and 7 being strongly agree.


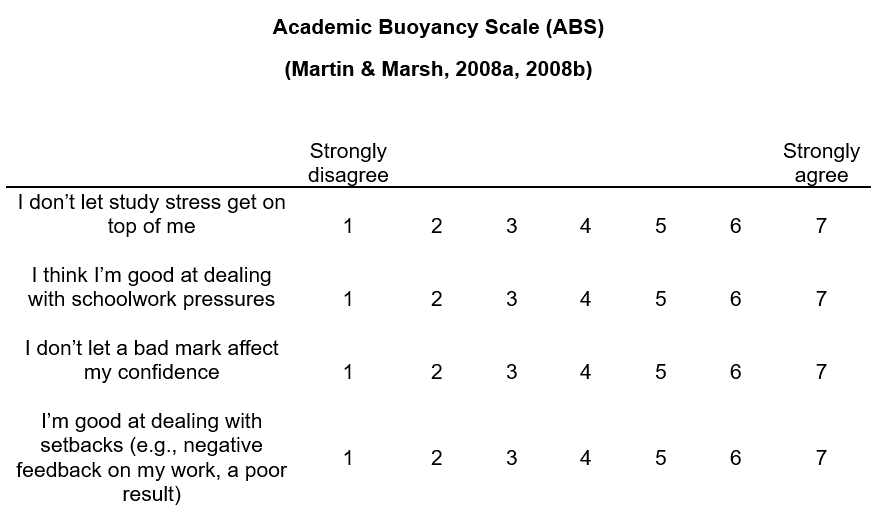


**Appendix 2 – Case profile and learning objectives**

Spontaneous secondary pneumothorax case profile and learning objectives outlined by Oxford Medical Simulation virtual reality software.

| Diagnosis | Spontaneous secondary pneumothorax |
| --- | --- |
| Case summary | Acutely tachypnoeic patient with pleuritic chest pain on a background of COPD. |
| Clinical findings | - Hypoxic, tachypnoeic, tachycardic - Hyper-resonant right hemithorax - Type 1 respiratory failure on ABG - Right-sided pneumothorax (<2cm) on CXR |
| Technical skills | Assessment   - Perform ABCDE assessment - Take focused patient history   Initial investigations   - Take blood tests - Take arterial blood gas - Order and review chest X-ray - Order ECG   Management   - Deliver oxygen to maintain saturations over 88% - Gain IV access - Administer analgesia - Insert chest drain under senior guidance - Repeat chest X-ray - Refer to appropriate team (respiratory) |
| Non-technical skills | - Make appropriate, timely decisions - Effectively communicate with patient - Effectively manage your team - Maintain patient safety and comfort - Access appropriate guidelines - Discuss case with seniors and respiratory team |

**Appendix 3 – VR scenario**

Example actions included conducting examinations (top left) and requesting investigations (top right, bottom left). Possible actions are displayed in a drop-down menu (bottom right).


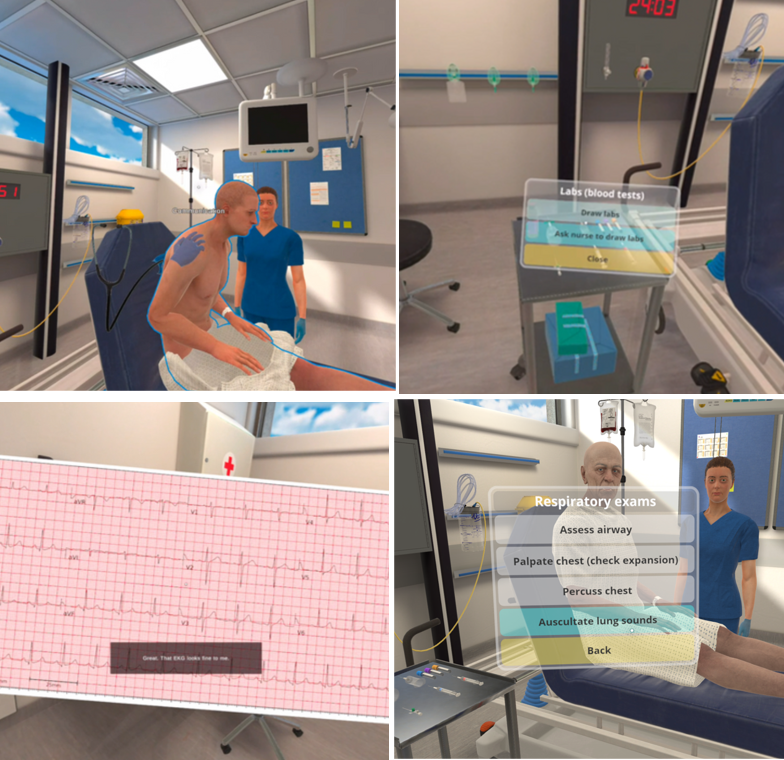


**Appendix 4 – Marking scheme**

Simulation marking scheme adapted using the virtual reality marking framework.

Acute surgical scenario simulation - Marking Scheme

Candidate name:

Marker:

| Initial  assessment | | Verify name and date of birth |  |
| --- | --- | --- | --- |
|  |  | Hand hygiene and PPE |  |
|  |  | Assess airway |  |
|  |  | Auscultate chest |  |
|  |  | Presenting complaint |  |
|  |  | Past medical history |  |
|  |  | Medication history |  |
|  |  | Allergies |  |
|  |  | Systems review |  |
|  |  | Cardiac exam |  |
|  |  | Examine legs (E.g., look for signs of swelling) |  |
| Investigations | | Obtain blood tests |  |
|  |  | Obtain arterial blood gas |  |
|  |  | Order and review chest X-ray |  |
|  |  | Order and review ECG |  |
| Management | | Continuous monitoring (E.g., ask nurse to apply continuous monitoring) |  |
|  |  | Deliver oxygen to maintain saturations over 88% |  |
|  |  | Gain IV access |  |
|  |  | Administer analgesia |  |
|  |  | Insert chest drain under senior guidance |  |
|  |  | Repeat chest X-ray |  |
|  |  | Consult with seniors and relevant team (E.g., Call for advice or to highlight an acutely unwell patient to them) |  |
|  |  | Refer to appropriate team (respiratory) |  |
|  |  | Update nurse |  |
| Additional | Efficient patient management (E.g. finish scenario in under 12 minutes) |  |  |
|  | Allocation of work (E.g. Ask nurse to help with taking bloods or inserting IV access) |  |  |
|  | Patient communication (E.g., Offer reassurance or update on care and progress) |  |  |
|  | Make sure of appropriate guidelines |  |  |

Score: …../28

**Appendix 5 – Actions categorised by skills domains**

List of possible actions to perform in the scenario, categorised by skills domains: communication, non-technical, teamwork, and technical.

| **SKILLS DOMAIN** | **ACTIONS** |
| --- | --- |
| **COMMUNICATION** | - Continuous monitoring - Presenting complaint - Past medical history - Medication history - Allergies - Phone respiratory team - Systems review - Patient communication - Verify name and date of birth |
| **NON-TECHNICAL** | - Phone senior - Patient communication - Update nurse - Verify name and date of birth |
| **TEAMWORK** | - Continuous monitoring - Oxygen delivery - Allocation of work - Phone senior - Update nurse |
| **TECHNICAL** | - Continuous monitoring - Oxygen delivery - Auscultate chest - Chest X-Ray - Presenting complaint - Past medical history - Blood tests - Analgesia administration - Insert chest drain - Medication history - Allergies - Arterial blood gas - Intravenous access - Electrocardiogram - Repeat Chest X-Ray - Assess airway - Phone respiratory team - Systems review - Cardiac exam - Access guidelines - Examine legs - Hand hygiene and personal protective equipment - Time efficiency |

**Appendix 6 – Graphical representations of OSCE scores and Skills domains**

Bar chart showing the comparison of mean post-session scores (%) between virtual reality and simulation groups. Note: an asterisk denotes p-value is significant, whereby * means p≤0.05.


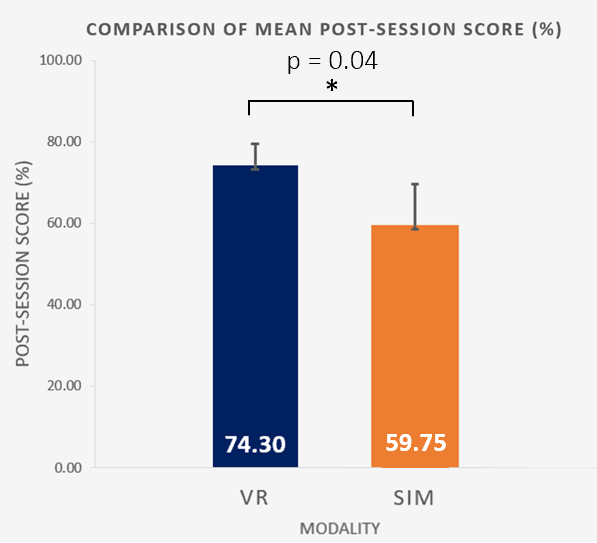


Bar charts showing the comparison of mean skills domains scores (%) between virtual reality and simulation groups. Note: an asterisk denotes p-value is significant, whereby ** means p≤0.01.


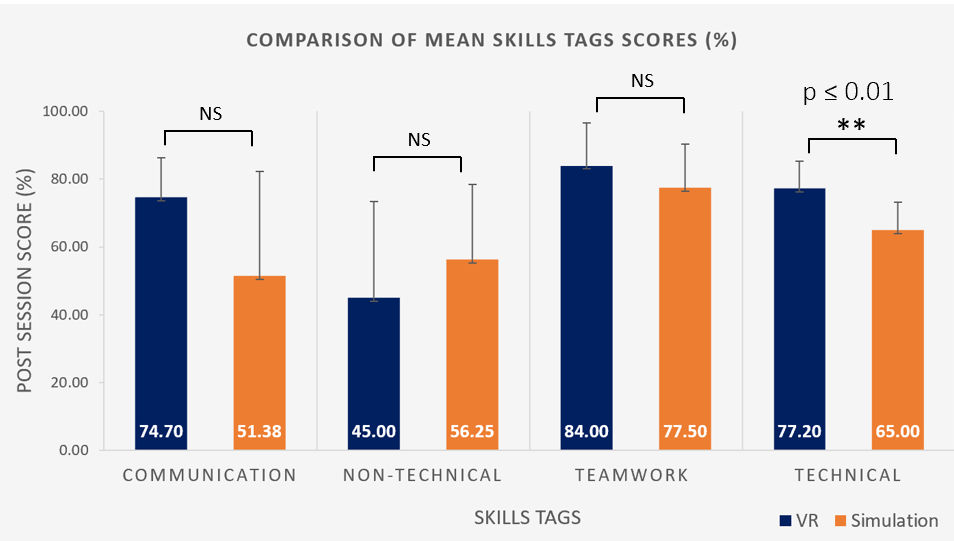


**Appendix 7 – Graphical representation of time-to-critical decision and completion**

Bar charts showing the comparison of mean time-to-critical decisions (min) between virtual reality and simulation groups. Note: an asterisk denotes p-value is significant, whereby ** means p≤0.01, and *** means p≤0.001.


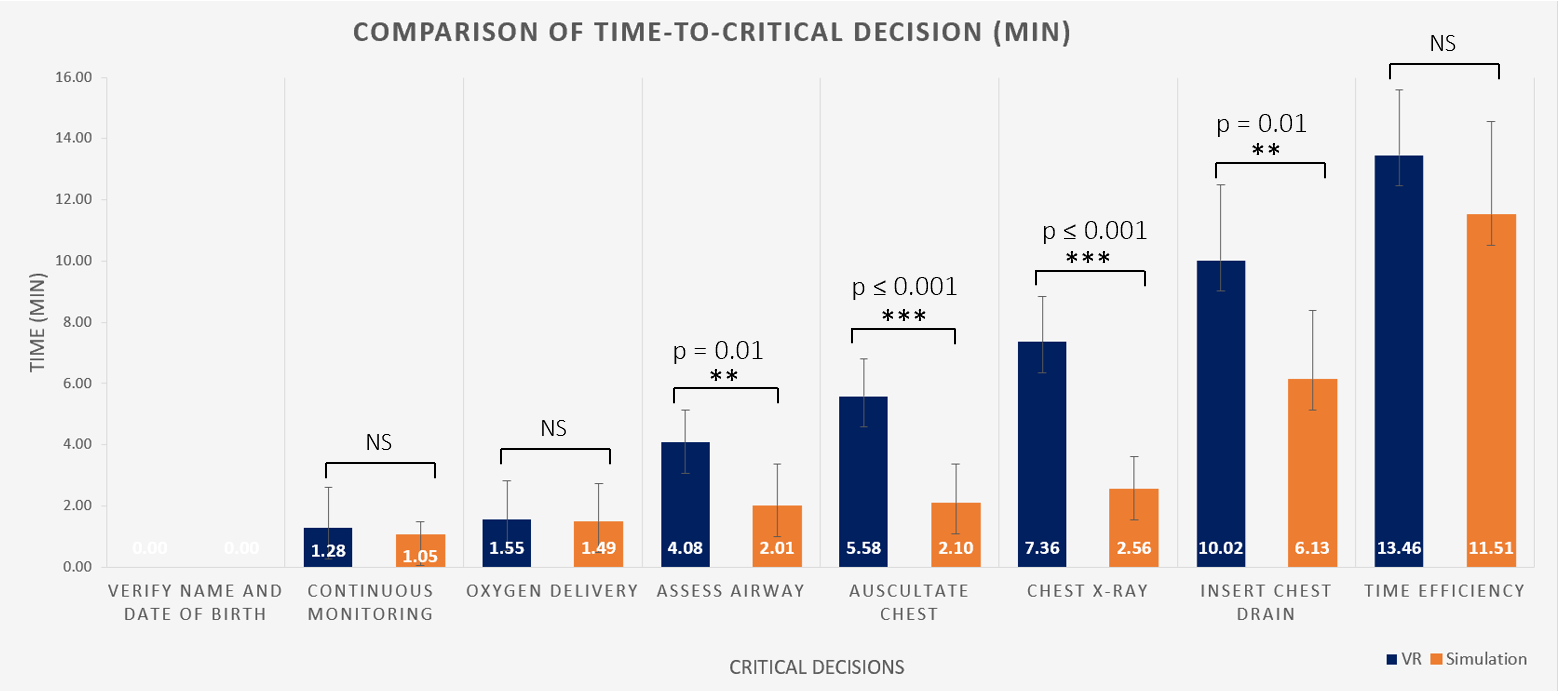


**Appendix 8 – Graphical representations of ABS scores**

Bar chart showing the comparison of mean pre- and post-ABS scores, of virtual reality and simulation groups. Note: an asterisk denotes p-value is significant, whereby ** means p≤0.01.

**
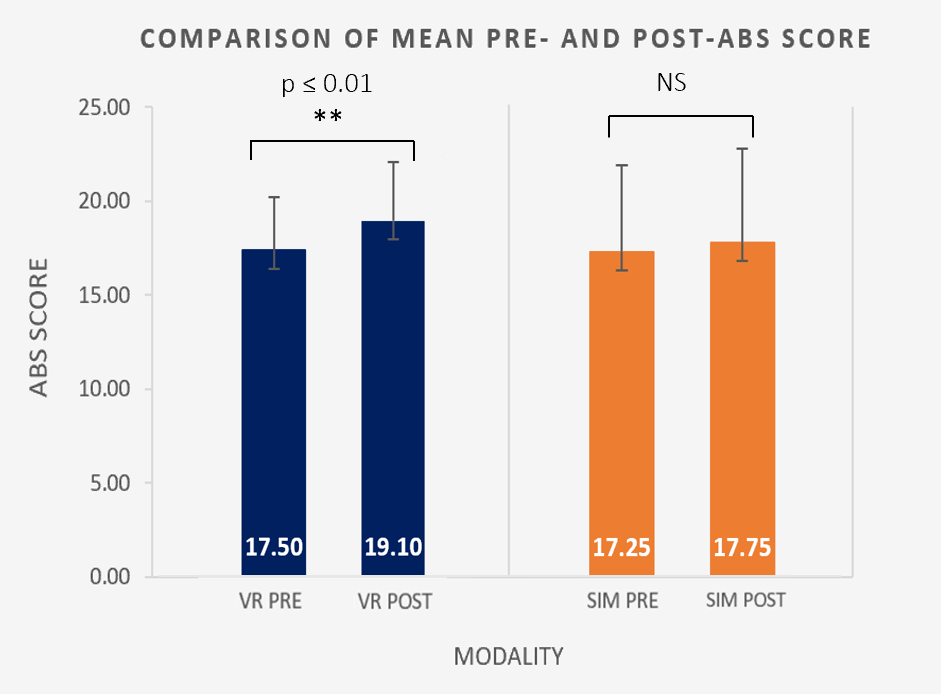
**

Scatter plot showing the comparison of OSCE and post-ABS scores.


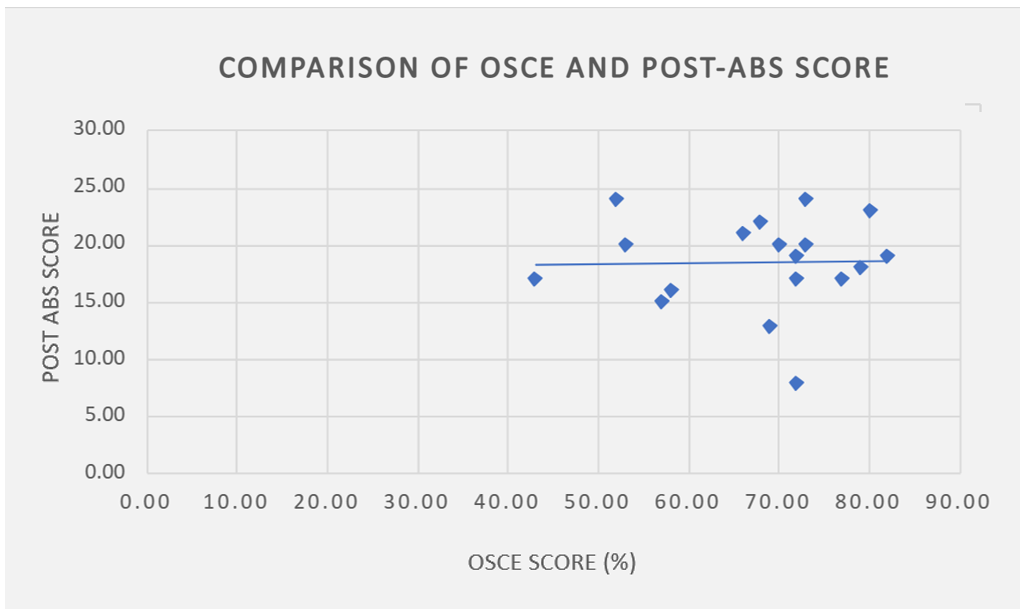

Supplement: Supplementary file 1 — Supporting information. [file HSR2-7-e2245-s001.docx]
